# Factors influencing the outcome of surgery for pelvic organ prolapse

**DOI:** 10.1007/s00192-017-3446-9

**Published:** 2017-09-11

**Authors:** Katja Stenström Bohlin, Maud Ankardal, Emil Nüssler, Håkan Lindkvist, Ian Milsom

**Affiliations:** 1 Department of Obstetrics and Gynecology, Sahlgrenska Academy at Gothenburg University, SE-416 85 Gothenburg, Sweden; 2 Department of Gynecology, Halland Hospital, Halmstad, Sweden; 3 Department of Obstetrics and Gynecology, Umeå University, Umeå, Sweden; 4 Department of Mathematics and Mathematical Statistics, Umeå University, Umeå, Sweden

**Keywords:** Body mass index, Mesh, Obesity, Pelvic organ prolapse, Postoperative complications, Urinary incontinence

## Abstract

**Introduction and hypothesis:**

Pelvic organ prolapse (POP) surgery is a common gynecological procedure. Our aim was to assess the influence of obesity and other risk factors on the outcome of anterior and posterior colporrhaphy with and without mesh.

**Methods:**

Data were retrieved from the Swedish National Register for Gynecological Surgery on 18,554 women undergoing primary and repeat POP surgery without concomitant urinary incontinence (UI) surgery between 2006 and 2015. Multivariate logistic regression analyses were used to identify independent risk factors for a sensation of a vaginal bulge, de novo UI, and residual UI 1 year after surgery.

**Results:**

The overall subjective cure rate 1 year after surgery was 80% (with mesh 86.4% vs 77.3% without mesh, *p* < 0.001). The complication rate was low, but was more frequent in repeat surgery that were mainly mesh related. The use of mesh was also associated with more frequent de novo UI, but patient satisfaction and cure rates were higher compared with surgery without mesh. Preoperative sensation of a vaginal bulge, severe postoperative complications, anterior colporrhaphy, prior hysterectomy, postoperative infections, local anesthesia, and body mass index (BMI) ≥30 were risk factors for sensation of a vaginal bulge 1 year postsurgery. Obesity had no effect on complication rates but was associated increased urinary incontinence (UI) after primary surgery. Obesity had no influence on cure or voiding status in women undergoing repeat surgery.

**Conclusions:**

Obesity had an impact on the sensation of a vaginal bulge and the presence of UI after primary surgery but not on complications.

## Introduction

The prevalence of pelvic organ prolapse (POP) is reported to be ≈ 10% [1–4]. Globally, up to half of all parous women have some degree of clinical prolapse, and 10–20% are symptomatic [1–4]. Vaginal childbirth and increasing parity are considered the strongest risk factors for the development of POP [4]. Other lifestyle factors, such as smoking and—in particular, obesity—are reported to be associated with the development of POP [4–7].

The lifetime risk of undergoing POP surgery alone varies between 5 and 19% [8]. The need for repeat POP surgery varies widely in the literature, but ~13% is an often-quoted figure and is even higher after an anterior colporrhaphy is performed [9, 10]. The use of mesh has shown improvement of anatomical and functional results compared with native tissue, but in recent years, alarming reports have arisen about increased complications due to mesh. New international guidelines recommend vaginal mesh repair to be performed by specialists and reserved for high-risk patients, such as women with recurrent prolapse [11]. Thus, in this respect, there is a need for continued evaluation of the outcome of POP surgery. There is also a need to further evaluate the impact of modifiable lifestyle factors, such as obesity, on the outcome of POP surgery, as there are few such evaluations at present, and the reported influence of obesity varies between studies [12–17]. Therefore, the aim of this study was to assess the influence of obesity and other risk factors on the outcome of anterior and posterior colporrhaphy with and without mesh.

## Materials and methods

This study is based on data from the Swedish National Register for Gynecological Surgery (GynOp, www.gynop.org) concerning women who underwent genital prolapse surgery between January 2006 and December 2015. To permit comparisons with earlier reports on comparable groups of patients undergoing POP surgery, we included women with simple anterior and/or posterior colporrhaphy only. Study participants were separated into primary and repeat surgery with or without mesh. The use of mesh in primary surgery is rare in Sweden, as is concomitant urinary incontinence (UI) surgery; therefore, those patients were excluded from the analyses. Repeat surgery was defined as being due to recurrence in the same compartment in accordance with earlier recommendations [10, 16]. Patients with repeat surgery in a different compartment was excluded.

### GynOp

The register was established in 1997, and prolapse surgery has been included since 2006, with 90% of clinics in Sweden providing information. Validated questionnaires concerning symptoms of prolapse and UI were used [18, 19]. Data was collected prospectively from patient questionnaires and doctors’ records. The patient received written information about the register and had the opportunity to decline participation. Preoperatively, the patient completed a questionnaire including a health declaration, subjective symptoms, and possible previous surgery. The surgeon registered data pertaining to preoperative findings and surgical history; information regarding a gynecological examination to assess prolapse stage; surgical procedure and postoperative events prior to discharge. Eight weeks and 1 year postoperatively, the patient received a questionnaire concerning complications, remaining prolapse-related symptoms such as the sensation of a vaginal bulge, questions on UI status, and satisfaction with surgery. The forms were evaluated by the surgeon if there were any complications and if so, whether they were minor or severe. A severe complication included those requiring a major intervention, such as organ lesions, excessive bleeding, deep venous thrombosis, or severe infection. To guide the surgeon when defining a severe complication, a guide is included in GynOp. A postoperative infection was recorded if the patient had received treatment with antibiotics due to surgical-site or urinary tract infection.

The absence of vaginal bulge symptoms is the strongest relationship with the patients’ assessment of overall improvement and treatment success after prolapse surgery [10]. Patients were considered “cured” if they never, hardly ever, 1–3 times per month had a bulging sensation 1 year after surgery. Question 16 in the Urogenital Distress Inventory (UDI) [20] was used for this purpose. In addition, information on patient satisfaction with surgery was obtained. Participants were asked preoperatively, 8 weeks, and 1 year after surgery how often they experienced urinary leakage and “troublesome UI” was indicated when experienced UI daily or more than one to three times a week.

### Statistical analyses

Continuous variables were analyzed using Student’s* t* test or analysis of variance (ANOVA), and categorical data were analyzed by Pearson’s chi-square test. Mann–Whithey* U* test was used to compare median values. A *p*value <0.05 was considered statistically significant. Multivariate logistic regression analysis models were constructed to assess risk factors for outcome variables while controlling for potential confounding factors. A stepwise approach was conducted to—one by one—exclude nonsignificant variables determined by multiple testing. Adjusted odds ratio (aOR) and the 95% confidence interval (CI) were calculated.

Factors that may contribute to complications, cure rate, and UI status were identified and included as confounders in statistical analysis; age, smoking, body mass index, previous hysterectomy, chronic constipation, ASA class, comorbidities (lung disease, diabetes and hypertension), preoperative prolapse stage > II, feeling of a bulging sensation daily or ≥1–3 times per week before surgery, prophylactic antibiotics, anesthetic type, type of surgery performed (anterior or posterior colporrhaphy) and the use of mesh. In the logistic regression analysis of subjective treatment success and UI the following additional variables were included: preoperative daily urgency, postoperative infection and severe complication.

Prior to conducting the logistic regression analysis, some of the independent variables were categorized. Body mass index (BMI) was divided into three groups according to the World Health Organization (WHO) classification: <25, 25–29.9, and ≥30. Based on information in the health declaration regarding smoking status, participants were classified as smoker or nonsmoker (includes former smokers). All statistical analyses were performed using SPSS version 21 or 23.

## Results

During the study period 15,833 women underwent primary surgery and 2721 repeat surgery due to POP relapse in the anterior and/or posterior compartment. Mesh-augmented surgery was performed in 1214/2721 (44.6%) of women undergoing repeat surgery.

Table 1 describes patient and surgery characteristics between primary and repeat surgery. Previous vaginal delivery was reported by >98% in both groups, and thus rates of nulliparous women or women delivered by cesarean section were extremely low in both groups. There were several differences between surgical groups: In repeat surgery, women were generaly older, had less hypertension and lung disease, and were more often classified as being American Society of Anesthesiology (ASA) class III–IV. The prevalence of being overweight but not of being obese was higher and smoking rate lower in the repeat-surgery group. Prior hysterectomy was more common among women undergoing repeat (27.5%) than those undergoing primary surgery (19.5%). Operating time and hospital stay were longer and peroperative bleeding higher in the repeat group. When subdividing the repeat-surgery group into mesh/no mesh, the reported adverse differences were driven by mesh.

**Table 1:** Characteristics of women undergoing primary and repeat prolapse surgery

	Primary surgery for rectocele and/or cystocele* n* = 15,833	Repeat surgery for rectocele and/or cystocele *n* = 2721
Age, mean (range)	63 (21–102)	66 (29-96)
Hypertension,* n* (%)	5163 (32.6)	802 (29.4)
Diabetes,* n* (%)	956 (6.0)	174 (6.4)
Lung disease,* n* (%)	2702 (19.1)	354 (13.0)
ASA class III–IV,* n* (%)	520 (3.3)	165 (6.1)
BMI, mean	26.2	26.5
<25,* n* (%)	4927 (35.7)	664 (31.1)
25–29,* n* (%)	6369 (46.1)	1044 (48.9)
≥30,* n* (%)	2508 (18.2)	427 (20)
Smoking,* n* (%)	1347 (9.5)	155 (8.1)
Previous hysterectomy,* n* (%)	2364 (19.5)	491 (27.1)
Parity, mean (range)	2.57 (0-12)	2.53 (0-11)
Prolapse stage >II,* n* (%)	3464 (29.5)	737 (34.7)
Bulging sensation preop,* n* (%)	12058 (86.9)	1884 (87.3)
Urinary incontinence preop,* n* (%)	5017 (35.7)	832 (38.1)
Prophylactic antibiotics ,* n* (%)	2666 (16.8)	1315 (48.4)
With mesh,* n* (%)	-	920 (75.8)
Without mesh,* n* (%)	-	395 (26.2)
Local anesthesia,* n* (%)	6801 (44.5)	675 (25.6)
General/regional anesthesia,* n* (%)	8470 (55.5)	1963 (74.4)
Colporrhaphy performed		
Anterior,* n* (%)	11,115 (70.2)	2235 (82.1)
Posterior,* n* (%)	6986 (44.1)	874 (32.1)
Mesh,* n* (%)	-	1214 (44.6)
Operating time (min), mean	43.1	48.2
Bleeding, ml, median	20	25
Hospital stay, days, mean	0.6	1.03

*ASA* American Society of Anesthesiologists,* BMI* body mass index

The response rate to the 8-week questionnaire was 86% and 74% for the 1-year questionnaire. In general, the degree of severe complications was low, but it was almost doubled in repeat surgery (Table 2). Postoperative infections and both severe and minor complications occurred more frequently in mesh surgery. In contrast, women who received mesh surgery reported the highest rates of satisfaction, improved condition, and lowest rates of sensation of vaginal bulge 1 year after surgery (Table 2). A description of registered peroperative complications, reported at discharge and from the 8-week questionnaire is presented in the “Appendix”.

**Table 2:** Peroperative complications, complication rates 8 weeks and 1 year after surgery, and subjective surgical outcome 1 year after surgery

	Primary surgery	vs	Repeat surgery	*p*-value	Repeat surgery			*p-*value
	*n* = 15 833		*n* = 2 721		No mesh	vs	Mesh	
					*n* = 1 507		*n* = 1 214	
At discharge								
Severe, n (%)	35 (0.3)		21 (0.8)	<0.001	7 (0.5)		14 (1.2)	0.05
Minor, n (%)	452 (2.9)		125 (4.6)	<0.001	46 (3.1)		79 (6.7)	<0.001
Complications 8 weeks postop								
Severe, n (%)	175 (1.2)		46 (1.9)	<0.001	19 (1.4)		27 (2.2)	0.14
Minor, n (%)	2083 (14.3)		402 (16.5)	<0.001	138 (10.3)		264 (23.5)	<0.001
Infection, n (%)	1151 (7.9)		213 (10.9)	0.11	91 (8.8)		122 (13.2)	0.001
Outcome 1-year postop								
No vaginal bulge, n (%)	8643 (80.2)		1461 (81.4)	<0.001	762 (77.3)		699 (86.4)	<0.001
Satisfaction with surgery, n (%)	8307 (75.9)		1344 (74.0)	<0.001	675 (68.2)		669 (81.0)	<0.001
Improved n (%)	8283 (84.2)		1170 (84.5)	0.49	576 (78.2)		594 (91.7)	<0.001
Neither improved nor worsened	1110 (11.3)		141 (10.2)	0.82	108 (14.7)		33 (5.1)	<0.001
Worsened	442 (4.5)		74 (5.3)	0.03	53 (7.2)		21 (3.2)	0.03

All complication variables were tested in relation to BMI and smoking, but no correlation was found. The feeling of a vaginal bulge showed a nonsignificant tendency to increase with increasing BMI, and less surgical satisfaction was reported by the higher BMI and smoker groups (data not shown). Several factors were identified on multivariate regression analysis for feeling a vaginal bulge 1 year after surgery and are presented in order of importance: preoperative sensation of vaginal bulge daily or ≥1–3 times/week, occurrence of severe postoperative complication, anterior colporrhaphy performed, prior hysterectomy, occurrence of postoperative infection, local anesthesia compared with general or regional anesthesia, and BMI ≥30 vs <25 (Table 3).

**Table 3:** Results of multivariate regression analysis of possible risk factors for feeling a vaginal bulge 1 year after primary surgery (*n* = 7209)

Variable	Crude OR (95% CI)	Adjusted OR (95% CI)
Age	1.00 (0.99–1.00)	1.00 (0.99–1.00)
Body mass index		
<25	1	1
25–29	1.08 (0.96–1.20)	1.13 (0.99–1.29)
≥30	1.13 (0.98–1.31)	1.20 (1.02–1.43)
Prior hysterectomy		
No	1	1
Yes	1.28 (1.12–1.45)	1.30 (1.15–1.53)
Preop sensation of vaginal bulge daily or 1–3 times/week		
No	1	1
Yes	2.76 (2.27–3.36)	2.66 (2.12–3.34)
Anesthesia		
General or regional	1	1
Local	1.26 (1.14–1.38)	1.26 (1.12–1.42)
Anterior colporrhaphy		
No	1	1
Yes	1.39 (1.25–1.56)	1.42 (1.23–1.63)
Complication 8 weeks		
Severe complication		
No	1	1
Yes	1.76 (1.15–2.69)	1.78 (1.06–2.98)
Infection		
No	1	1
Yes	1.29 (1.09–1.53)	1.31 (1.07–1.61)

ASA class, hypertension, diabetes, lung disease, chronic constipation, smoking, preoperative prolapse stage > II, parity, and posterior colporrahpy initially included were nonsignificant

*OR* odds ratio,* CI* confidence interval,* ASA* American Society of Anesthesiologists

Preoperative UI was more common in women undergoing repeat than primary surgery (Table 1). Residual and de novo UI occurred more often after repeat surgery and was most pronounced in the mesh group. Up to 49% had UI remission after surgery (Table 4). Remission and residual UI rates at 8 weeks did not differ after 1 year, but de novo UI was less prominent after 1 year following repeat surgery with mesh (Table 4). Increasing BMI was correlated with an increase in de novo and residual UI and a decrease in UI remission following primary surgery. Trends for BMI following repeat surgery were seen but were not statistically significant (Table 5).

**Table 4:** Preoperative remission, residual, and de novo urinary incontinence (UI) in women following primary and repeat surgery with and without mesh 8 weeks and 1 year after surgery

	Primary surgery [*n* (%)]	Repeat surgery without mesh [*n* (%)]	*P* value, primary vs repeat surgery without mesh	Repeat surgery with mesh [*n* (%)]	*P* value Repeat surgery, no mesh vs mesh
Preoperative	5017 (35.7)	464 (37.3)	0.27	368 (39.2)	0.37
Remission of					
8 weeks	2136 (48.8)	172 (42.7)	0.02	136 (40.1)	0.50
1 year	1661 (47.9)	136 (40.8)	0.02	111 (44.0)	0.45
Residual					
8 weeks	2241 (51.2)	231 (57.3)	0.02	203 (55.2)	0.56
1 year	1810 (52.1)	197 (59.2)	0.01	141 (56.0)	0.45
De novo					
8 weeks	828 (10.3)	82 (12.3)	0.01	121 (23.9)	<0.001
1 year	703 (10.5)	83 (14.9)	0.002	75 (13.1)	0.39

**Table 5:** Association between body mass index (BMI) and remission, de novo, and residual urinary incontinence (UI) in primary and repeat pelvic organ prolapse POP surgery

8 weeks postop	Primary, [*n* (%)]	*P* value	Repeat, [*n* (%)]	*P* value
Remission				
BMI <25	677 (53.5)	<0.001	82 (45.1)	0.13
25–29	978 (49.2)		152 (43.2)	
≥30	398 (42.8)		59 (35.1)	
Residual				
BMI <25	588 (46.5)	<0.001	100 (54.9)	0.13
25–29	1010 (50.8)		200 (56.8)	
≥30	532 (57.2)		109 (64.9)	
De novo				
BMI <25	251 (8.3)	<0.001	68 (17.6)	0.82
25–29	383 (10.7)		88 (16.4)	
≥30	161 (13.5)		35 (18.2)	

Multivariate regression analysis identified increasing age [aOR 1.01; 95% CI1.01–1.02), overweight (aOR 1.18; 95% CI 1.02–1.36), and obesity (aOR 1.49; 95% CI1.25–1.77) to be predictors of residual UI. The risk of residual UI was lower (aOR 0.56; 95%CI 0.48–0.65) in women who underwent anterior colporrhaphy compared with those who did not (data not shown).

## Discussion

The outcome of POP surgery was reviewed by Maher et al. [21], who reported cure rates following anterior colporrhaphy between 37 and 97% and for posterior colporrhaphy between 56 and 100%. In this national cohort of women, cure—as defined by the absence of a sensation of a vaginal bulge 1 year after surgery—was 80% for primary and 81% for repeat surgery. Women undergoing repeat surgery with mesh reported a higher success rate compared with women without mesh. There were, however, more postoperative infections and severe and minor complications recorded in women undergoing repeat surgery with mesh.

This study also identified several independent risk factors for the sensation of a vaginal bulge 1 year after surgery: daily or ≥1–3 times/week sensation preoperatively predicted noncure. It is highly likely that frequent sensation preoperatively is associated with the degree of prolapse, and Salvatore et al. previously reported that a preoperative prolapse stage ≥III is a significant risk factor for recurrence [22]. Sensation frequency experienced prior to surgery may thus be a potential indicator for subsequent surgical success.

Hysterectomy is associated with an increased risk of POP [4]. In this study, prior hysterectomy was an independent risk factor for noncure following prolapse surgery. Previous studies indicate that hysterectomy due to prolapse—rather than hysterectomy per se—increases the risk of subsequent prolapse surgery [23, 24]. Hysterectomy is also a proposed risk factor for the development of UI [25–27], but in our analysis of factors contributing to UI after prolapse surgery, prior hysterectomy was not a predictor. These results illustrate the complexity of pelvic floor disorders.

Obesity was an independent risk factor for POP surgery failure. Women with a BMI ≥ 30 undergoing primary surgery more frequently felt a vaginal bulge, reported de novo UI, and experienced residual UI and less remission 1 year postsurgery. Obesity has previously been identified as an important risk factor for UI after POP surgery [28]. In contrast, obesity did not influence the risk of complications, and there was no difference in minor or severe complication rates recorded in obese women. Our findings are in agreement with a retrospective study that found no effect of being overweight on perioperative complications, including hospital stay, after vaginal surgery [15].

Even though obese women had an increased risk of subjective symptoms of POP recurrence, the proportion of obese women undergoing repeat surgery was not influenced. Nor did obesity influence main outcome variables, cure rate, or UI remission, residual UI, and de novo UI rates in women undergoing repeat surgery. One possible explanation is that obese women after primary prolapse surgery chose to undergo stress UI (SUI) surgery, making it less likely they will choose a third surgical intervention despite remaining prolapse symptoms. It might also be that surgeons are more reluctant to perform repeat surgery on obese women to avoid further failure.

In previous reports smokers have a higher risk of mesh erosions, which are unfortunately not specified in GynOp. However, there was no increased rate among smokers of dyspareunia or reoperation at 1 year after surgery. Interesting to note, >98% of the women had a vaginal delivery, and only 0.7% were nulliparous. The low percentage of nulliparous women undergoing prolapse surgery is in strong contrast to the total number of such women who are resident in Sweden aged 65 years, with a mean (range) of 13.2% (12.3–14.1).

The main strengths of this study are the use of a national database consisting of a large, unselected patient population with a wide variety of variables, and the high response rate at the 1-year follow-up (74%). Patient acceptance of questionnaires for gathering information regarding gynecological procedures reported to the GynOp was evaluated previously, well accepted by patients, and provided complete posttreatment information [29]. One possible limitation of large register studies is the lack of verification of objective surgical success, whether the bulging sensation indicates a relapse or not, and—in particular—whether relapse is in the same compartment. However, previous studies show that a simple question regarding feeling of a vaginal bulge can accurately screen for POP, without the need for a physical examination [18, 30].

The overall cure rate 1 year after POP surgery was 80% (with mesh 86% vs 77% without mesh). The rate of severe complications was low for both primary and repeat POP surgery. Preoperative sensation of a vaginal bulge daily to ≥ 1–3 times/week, severe postoperative complication, anterior vs posterior colporrhaphy, prior hysterectomy, postoperative infection, local vs general or regional anesthesia, and BMI ≥30 were independent risk factors for the sensation of a vaginal bulge 1 year after primary surgery. Obesity had an impact on subjective success and voiding function but not on complication rate. There was, however, no influence of obesity on outcome in women undergoing repeat surgery. Several of the risk factors identified are modifiable and thus of importance when contemplating prolapse surgery and in connection with the provision of information to the patient prior to surgery.

## Appendix

**Table 6:** Specified complications in primary pelvic organ prolapse surgery

Complications	Severity	Peroperative (*n* = 15,824)	At discharge (*n* = 15,296)	8-week questionnaire (*n* = 14,540)
Overall	Minor	91 (0.6)	452 (2.9)	2083 (14.3)
	Severe	19 (0.1)	35 (0.3)	175 (1.2)
Bleeding	Minor	51 (0.3)	135 (0.9)	376 (2.6)
	Severe	12 (0.1)	18 (0.1)	53 (0.4)
Organ lesions				
Intestinal	Minor	12 (0.1)	7 (0.05)	159 (1.0)
	Severe	7 (0.04)	1 (0.01)	31 (0.2)
Uterus	Minor	1 (0.01)	-	28 (0.2)
	Severe	1 (0.01)	-	11 (0.07)
Vagina	Minor	-	-	446 (2.9)
	Severe	-	-	39 (0.3)
Bladder	Minor	23 (0.1)	25 (1.6)	277 (1.8)
	Severe	10 (0.1)	3 (0.02)	25 (0.2)
Ureter	Minor	1 (0.01)		12 (0.08)
	Severe	3 (0.02)	1 (0.01)	3 (0.02)
Urethra	Minor	1 (0.01)	-	144 (0.9)
	Severe	4 (0.03)	-	13 (0.1)
Other	Minor	7 (0.04)	8 (0.05)	233 (1.5)
	Severe	2 (0.01)	1 (0.01)	32 (0.3)
Urinary retention		-	154 (1.1)	186 (1.2)
Infections				
Urinary tract infection	Minor	-		451 (3.0)
	Severe	-	60 (0.4)	22 (0.1)
Wound	Minor	-	21 (0.1)	360 (2.5)
	Severe	-	13 (0.1)	36 (0.2)
Sepsis		-	1 (0.01)	10 (0.1)
Other infection	Minor	-	-	75 (0.5)
	Severe	-	-	13 (0.1)
Deep venous thrombosis		-	-	9 (0.06)
Fistula	Minor	-	-	11 (0.1)
	Severe	-	-	5 (0.03)

**Table 7:** Specified complications in repeat pelvic organ prolapse surgery with mesh

Complications	Severity	Peroperative (*n* = 1214)	At discharge (*n* = 1183)	8-week questionnaire (*n* = 1153)
Overall	Minor	23 (1.9)	79 (6.7)	264 (23.5)
	Severe	5 (0.4)	14 (1.2)	27 (2.2)
Bleeding	Minor	10 (0.8)	9 (0.8)	23 (2.0)
	Severe	2 (0.2)	4 (0.3)	5 (0.4)
Organ lesions				
Intestinal	Minor	-	-	12 (1.0)
	Severe	-	-	3 (0.3)
Uterus	Minor	-	-	-
	Severe	-	-	1 (0.1)
Vaginal	Minor	-	-	33 (2.9)
	Severe	-	-	2 (0.2)
Bladder	Minor	11 (0.9)	10 (0.8)	39 (3.2)
	Severe	4 (0.3)	6 (0.5)	10 (0.8)
Ureter	Minor	-	-	2 (0.2)
	Severe	-	-	1 (0.1)
Urethra	Minor	-	-	17 (1.5)
	Severe	-	1 (0.1)	3 (0.3)
Other	Minor	3 (0.2)	-	29 (2.5)
	Severe	-	-	4 (0.3)
Urinary retention		-	31 (2.6)	25 (2.2)
Infections				
Urinary tract infection				
	Minor	-	12 (1.0)	73 (6.3)
	Severe	-	-	6 (0.5)
Wound	Minor	-	4 (0.3)	29 (2.5)
	Severe	-	1 (0.1)	12 (1.0)
Sepsis		-	-	2 (0.2)
Other infection	Minor	-	3 (0.3)	8 (0.7)
	Severe	-	-	2 (0.2)
Deep venous thrombosis		-	-	1 (0.1)
Fistula	Minor	-	-	-
	Severe	-	-	1 (0.1)

**Table 8:** Specified complications in repeat surgery without mesh

Complications	Severity	Peroperative (*n* = 1506)	At discharge (*n* = 1485)	8-week questionnaire (*n* = 1271)
Overall	Minor	46 (3.1)	46 (3.1)	138 (10.3)
	Severe	3 (0.2)	7 (0.5)	19 (1.4)
Bleeding	Minor	3 (0.2)	8 (0.5)	32 (2.5)
	Severe	1 (0.1)	-	8 (0.6)
Organ lesions				
Intestinal	Minor	1 (0.1)	-	11 (0.8)
	Severe	1 (0.1)	-	2 (0.1)
Uterus	Minor	-	-	1 (0.1)
	Severe	-	-	-
Vaginal	Minor	-	-	32 (2.3)
	Severe	-	-	4 (0.3)
Bladder	Minor	6 (0.4)	6 (0.4)	26 (1.9)
	Severe	10 (0.7)	5 (0.3)	6 (0.5)
Ureter	Minor	1 (0.1)	-	-
	Severe	-	-	2 (0.1)
Urethra	Minor	-	-	15 (1.1)
	Severe	1 (0.1)	-	1 (0.1)
Other	Minor	1 (0.1)	3 (0.2)	16 (1.2)
	Severe	-	-	1 (0.1)
Urinary retention	Minor	-	18 (1.2)	17 (1.2)
Infections				
Urinary tract infection				
	Minor	-	3 (0.2)	40 (2.9)
	Severe	-	1 (0.1)	5 (0.3)
Wound	Minor	-	1 (0.1)	24 (1.9)
	Severe	-	-	4 (0.3)
Sepsis		-	-	-
Other infection	Minor	-	-	1 (0.1)
	Severe	-	-	1 (0.1)
Deep venous thrombosis		-	-	-
Fistula	Minor	-	-	3 (0.2)
	Severe	-	-	2 (0.1)

## References

[1] Bradley CS, Zimmerman MB, Qi Y, Nygaard IE (2007). Natural history of pelvic organ prolapse in postmenopausal women. Obstet Gynecol.

[2] Rortveit G, Brown JS, Thom DH, Van Den Eeden SK, Creasman JM, Subak LL (2007). Symptomatic pelvic organ prolapse: prevalence and risk factors in a population-based, racially diverse cohort. Obstet Gynecol.

[3] Gyhagen M, Bullarbo M, Nielsen TF, Milsom I (2013). Prevalence and risk factors for pelvic organ prolapse 20 years after childbirth: a national cohort study in singleton primiparae after vaginal or caesarean delivery. BJOG.

[4] Milsom I AD, Cartwright R, Lapitan MC, Nelson R, Sillén U, Tikkanen K (2013) Epidemiology of Urinary Incontinence (UI) and other Lower Urinary Tract Symptoms (LUTS), Pelvic Organ Prolapse (POP) and Anal (AI) Incontinence. 5 th International Consultation on Incontinence edn.

[5] Hendrix SL, Clark A, Nygaard I, Aragaki A, Barnabei V, McTiernan A (2002). Pelvic organ prolapse in the Women's Health Initiative: gravity and gravidity. Am J Obstet Gynecol.

[6] Kudish BI, Iglesia CB, Sokol RJ, Cochrane B, Richter HE, Larson J, Hendrix SL, Howard BV (2009). Effect of weight change on natural history of pelvic organ prolapse. Obstet Gynecol.

[7] Chen CC, Gatmaitan P, Koepp S, Barber MD, Chand B, Schauer PR, Brethauer SA (2009). Obesity is associated with increased prevalence and severity of pelvic floor disorders in women considering bariatric surgery. Surg Obes Relat Dis : Off J Am Soc Bariatric Surg.

[8] Haya N, Baessler K, Christmann-Schmid C, de Tayrac R, Dietz V, Guldberg R, Mascarenhas T, Nussler E, Ballard E, Ankardal M, Boudemaghe T, Wu JM, Maher CF (2015). Prolapse and continence surgery in countries of the Organization for Economic Cooperation and Development in 2012. Am J Obstet Gynecol.

[9] Clark AL, Gregory T, Smith VJ, Edwards R (2003). Epidemiologic evaluation of reoperation for surgically treated pelvic organ prolapse and urinary incontinence. Am J Obstet Gynecol.

[10] Barber MD, Maher C (2013). Epidemiology and outcome assessment of pelvic organ prolapse. Int Urogynecol J.

[11] Administration UFaD (2013) FDA public healt notification: serious complications associated with transvaginal placement of surgical mesh in repair of pelvic organ prolapse and stress urinary incontinence. www.fdagov/MedicalDevices/Safety/AlertsandNotices/PublicHelathNotifications/ucm061976htm. 10.1016/j.eururo.2009.01.055 19645092

[12] Kawasaki A, Corey EG, Laskey RA, Weidner AC, Siddiqui NY, Wu JM (2013). Obesity as a risk for the recurrence of anterior vaginal wall prolapse after anterior colporrhaphy. J Reprod Med.

[13] Greer WJ, Richter HE, Bartolucci AA, Burgio KL (2008). Obesity and pelvic floor disorders: a systematic review. Obstet Gynecol.

[14] Tuschy B, Berlit S, Kehl S, Sutterlin M, Bussen S (2012). Influence of overweight in elderly patients undergoing vaginal surgery due to pelvic floor disorders. Vivo.

[15] Haverkorn RM, Williams BJ, Kubricht WS 3rd, Gomelsky A. Is obesity a risk factor for failure and complications after surgery for incontinence and prolapse in women? J Urol. 185(3):987–92. 10.1016/j.juro.2010.10.064 21247603

[16] Maher C, Feiner B, Baessler K, Schmid C (2013) Surgical management of pelvic organ prolapse in women. Cochrane Database Syst Rev (4):CD004014. doi:10.1002/14651858.CD004014.pub5. 10.1002/14651858.CD004014.pub5 23633316

[17] Lo TS, Tan YL, Khanuengkitkong S, Dass AK (2013). Surgical outcomes of anterior trans-obturator mesh and vaginal sacrospinous ligament fixation for severe pelvic organ prolapse in overweight and obese Asian women. Int Urogynecol J.

[18] Tegerstedt G, Miedel A, Maehle-Schmidt M, Nyren O, Hammarstrom M (2005). A short-form questionnaire identified genital organ prolapse. J Clin Epidemiol.

[19] Kulseng-Hanssen S, Borstad E (2003). The development of a questionnaire to measure the severity of symptoms and the quality of life before and after surgery for stress incontinence. BJOG.

[20] Shumaker SA, Wyman JF, Uebersax JS, McClish D, Fantl JA (1994). Health-related quality of life measures for women with urinary incontinence: the incontinence impact questionnaire and the Urogenital distress inventory. Continence program in women (CPW) research group. Qual Life Res.

[21] Maher C BK, Barber M, Cheon C, Deitz V, De Tayrac R, Gutman R, Sentilhes L, Karram M. (2013) Pelvic organ prolapse surgery. 5th edition edn.

[22] Salvatore S, Athanasiou S, Digesu GA, Soligo M, Sotiropoulou M, Serati M, Antsaklis A, Milani R (2009). Identification of risk factors for genital prolapse recurrence. Neurourol Urodyn.

[23] Dallenbach P, Kaelin-Gambirasio I, Dubuisson JB, Boulvain M (2007). Risk factors for pelvic organ prolapse repair after hysterectomy. Obstet Gynecol.

[24] Lykke R, Blaakaer J, Ottesen B, Gimbel H (2015). The indication for hysterectomy as a risk factor for subsequent pelvic organ prolapse repair. Int Urogynecol J.

[25] Bohlin KS, Ankardal M, Lindkvist H, Milsom I (2017). Factors influencing the incidence and remission of urinary incontinence after hysterectomy. Am J Obstet Gynecol.

[26] Altman D, Granath F, Cnattingius S, Falconer C (2007). Hysterectomy and risk of stress-urinary-incontinence surgery: nationwide cohort study. Lancet.

[27] Andersen LL, Moller LM, Gimbel H (2015). Lower urinary tract symptoms after subtotal versus total abdominal hysterectomy: exploratory analyses from a randomized clinical trial with a 14-year follow-up. Int Urogynecol J.

[28] Lensen EJ, Withagen MI, Kluivers KB, Milani AL, Vierhout ME (2013). Urinary incontinence after surgery for pelvic organ prolapse. Neurourol Urodyn.

[29] Ladfors MB, Lofgren ME, Gabriel B, Olsson JH (2002). Patient accept questionnaires integrated in clinical routine: a study by the Swedish National Register for gynecological surgery. Acta Obstet Gynecol Scand.

[30] Barber MD, Neubauer NL, Klein-Olarte V (2006). Can we screen for pelvic organ prolapse without a physical examination in epidemiologic studies?. Am J Obstet Gynecol.

